# Prednisone may rebuild the immunologic homeostasis: Alteration of Th17 and Treg cells in the lymphocytes from rats' spleens after treated with prednisone‐containing serum

**DOI:** 10.1002/mgg3.800

**Published:** 2019-06-06

**Authors:** Xiao‐Qian Fu, Jun‐Ying Cai, Mu‐Jun Li

**Affiliations:** ^1^ Department of Reproductive Center First Affiliated Hospital of Guangxi Medical University Nanning China; ^2^ Department of Reproductive Center The Maternal and Child Health Hospital, The Obstetrics and Gynecology Hospital of Guangxi Zhuang Autonomous Region Nanning Guangxi China

**Keywords:** immunologic homeostasis, prednisone, Th17 cells, Treg cells

## Abstract

**Background:**

This study aimed to investigate alterations of T helper 17 (Th17), regulatory T (Treg) cells and relative cytokines after treating with prednisone‐contained serum. Lymphocytes were isolated from female rats' spleens.

**Methods:**

The splenic lymphocytes were divided into four groups: which were treated with normal rats' serum (control); prednisone‐containing rats' serum (PDN); normal rats' serum and cytokines (CTK); cytokines and prednisone‐containing rats' serum (PDN + CTK). The mRNA expression level of RORC, Foxp3 and interleukin‐17 (IL‐17) was examined by reverse transcription‐polymerase chain reaction. The quantities of Th17 and Treg cells were tested by flow cytometry, and the concentrations of IL‐17 and IL‐10 were detected by enzyme‐linked immunosorbent assay.

**Results:**

Higher mRNA expression level of Foxp3, percentages of Treg/CD4^+^, and concentrations of IL‐10, lower mRNA expressions of RORC and IL‐17, concentrations of IL‐17 and percentages of Th17/CD4^+^ in PDN group were detected, compared with control group (all *p* < 0.01). Similar trend was detected in PDN + CTK group, compared with CTK group (all *p* < 0.01).

**Conclusion:**

Our results suggest that prednisone may rebuild the immunologic homeostasis and may be used in human diseases with changes in the imbalance immune system such as unexplained recurrent spontaneous abortion (URSA), hepatitis B infection, or other autoimmune diseases.

## 1 INTRODUCTION

Prednisone is a synthetic corticosteroid drug which is particularly active as an immunosuppressant drug. It can restrain the hyperplasia of connective tissue, reduce the permeability of capillary wall and cell membrane, reduce inflammatory exudation, and inhibit the production and release of histamine and other inflammatory mediators. It is used to treat some inflammatory diseases and certain autoimmune diseases. In recent years, researchers have used this drug to treat some diseases which were not considered to be immune diseases, such as unexplained recurrent spontaneous abortions (URSA) (Han et al., 2012), repeated implantation failure (RIF) (Nyborg, Kolte, Larsen, & Christiansen, 2014), hepatitis B (Lu, Huang, Sun, Zhu, & Cui, 2014), and some kinds of cancer (Chen et al., (2013). Although these studies have demonstrated the effectiveness of prednisone, the pharmacological mechanism of this drug in regulation of immunologic homeostasis has not been completely clarified.

CD4^+^ cells play a key role in the maintenance of host immune balance, such as preventing infection, inhibiting tumor growth, limiting inflammation, and autoimmunity. Besides the typical Th (T helper) cells, Th1 and Th2, Th17 cells which express IL‐17 have been shown to promote inflammation and host defense against extracellular pathogens by mediating the supplement of neutrophils and macrophages to infected tissues. It has been confirmed that the percentage of Th17 cells was higher in the peripheral blood of patients with URSA, suggesting that Th17 cells play a potentially negative role in the maintenance of pregnancy (Liu et al., (2011). CD4^+^ regulatory T cells (Tegs) that express the transcription factor Foxp3 are regarded to be essential for keeping CD4^+^ effector T cells in charge. Jasper, Tremellen, and Robertson (2006) shown that the mRNA expression of Foxp3 was obviously reduced in the endometrium tissue of infertile women, particularly in those experiencing repeated failed cycles of in vitro fertilization. Lee et al. (2011) reported that the imbalance of Th17/Treg cells and the consequent changes in cytokine expression were correlated with the pathogenesis of URSA. Furthermore, literature indicated that lymphocyte immunization therapy could affect the Th17/Treg balance and be beneficial to pregnancy (Wu et al., 2014). In patients with severe hepatitis B, glucocorticoid treatment could prevent the progression and the patients’ condition can be cured (Lu et al., 2014). Chen et al. (2013) reported that the Th17/Treg balance and the expression of related cytokines are abnormal in cervical cancer patients.

It is known to all that CD4^+^ T cells will differentiate into Th17 cells in the presence of interleukin‐6 (IL‐6) and transforming growth factor‐beta (TGF‐β) or differentiate into Treg cells in the absence of IL‐6 and the presence of TGF‐β (Saifi et al., 2014). In addition, interleukin‐23 (IL‐23) also involves in maintenance and differentiation of lymphocytes into Th17 cells (Cai & Li, 2016). In the current study, we induced the lymphocytes which were separated from the rats' spleens differentiation into Th17 cells with the effects of IL‐23, IL‐6, and TGF‐β to simulate the microenvironment of immunologic rejection. Then, we evaluated whether the prednisone‐containing serum could modulate the balance of Th17/Treg cells and related cytokines.

## 2 MATERIALS AND METHODS

### 2.1 Animals and Materials

Female Wistar rats (*n* = 40, body weight 220 ± 10 g, mean age 12 weeks) were obtained from the animal experimental center of Guangxi Medical University (China). Animals were housed at 20°C–25°C and 50 ± 5% humidity with ad libitum access to food and water and a 12:12 hr light/dark cycle. This study was approved by the Medical Ethics Committee of First Affiliated Hospital of Guangxi Medical University. The animals were prepared for isolation of spleen lymphocytes and preparation of prednisone‐containing serum.

Prednisone was purchased from Xianju pharmaceutical factory (Zhejiang, China). The anti‐CD3 antibody, IL‐6, IL‐23, and TGF‐β were purchased from eBioscience (California, USA). Culture medium and fetal bovine serum were purchased from Gibco (California, USA). PrimeScript RT reagent kit was purchased from Baosheng (Dalian, China). All PCR primers were designed to flank the exon–intron boundaries to avoid amplification of genomic sequences and synthesized by Huada (Beijing, China). The antibody for flow cytometry, PerCP‐Cy5.5‐conjugated anti‐CD4, phycoerythrin (PE)‐conjugated anti‐human IL‐17, APC‐conjugated anti‐CD25, and Alexa Fluor488‐conjugated anti‐CD127 were provided by Becton Dickinson (New Jersey, USA). Enzyme‐linked immunosorbent assay (ELISA) kits were purchased from Cusabio (Wuhan, China).

### 2.2 Preparation of prednisone‐containing serum

Serum preparation rats were divided into two groups (*n* = 10 for each group): control group and prednisone‐treated group. The rats in prednisone‐treated group received prednisone solution in the dose of 2.5 mg/kg body weight/day by gavage once every day for 3 days. On the fourth day, the animals were fasted and received prednisone solution in the dose of 2.5 mg/kg body weight twice (with an interval of more than 2 hr). In contrast, the rats in the control group received an equivalent volume of normal saline. All the rats were anesthetized by 10% chloral hydrate (Sangon, China) injection administered intraperitoneally at 0.5 ml/100 g body weight. Blood was extracted from aorta abdominalis, and then centrifuged at 400 *g* for 15 min. The supernatant serum was collected, sterilized by 0.22 μm sterilizing filter, inactivated at 56°C for 30 min, then stored at −80°C. Given that the prednisone was transformed into prednisolone in hepatic metabolism, we performed high performance liquid chromatography to detect the serum concentration of prednisolone. In the prednisone‐treated group, the serum level of prednisolone is 7.63 ± 1.64 μg/ml (mean ± *SD*). Prednisolone was not detected in the serum of the control group.

### 2.3 Isolation and cultivation of the spleen lymphocytes

Twenty rats were anesthetized by 10% chloral hydrate injection administered intraperitoneally at 0.5 ml/100 g body weight. Subsequently, laparotomy of all rats was performed; spleen was aseptically obtained and rinsed with cold phosphate‐buffered saline (PBS, pH 7.2, Solarbio, China). The spleen was further cut into slices and pressed through 70 μm colatorium to RPMI‐1640 medium. Then the suspension was centrifuged at 400 *g* in lymphocytes separation medium. The spleen lymphocytes were resuspended in DMEM/F12 supplemented with 10% fetal bovine serum, 100 U/ml penicillin and 100 mg/ml streptomycin (Invivogen, USA).

### 2.4 Treatment of the spleen lymphocytes

Spleen lymphocytes were seeded into 6‐well plates (2 × 10^6^/well) that were precoated with 10 μg/ml purified anti‐CD3 antibody overnight at 4°C. To examine the effect of prednisone‐containing serum on spleen lymphocytes, the lymphocytes were divided into four groups: which were further treated for 72 hr with 1 μmol/L normal rats' serum (control); with 1 μmol/L prednisone‐containing rats' serum (PDN); with 1 μmol/L normal rats' serum and 10 ng/ml IL‐23, 20 ng/ml IL‐6, and 5 ng/ml TGF‐β1 as proinflammatory cytokines (CTK); or the three proinflammatory cytokines for 24 hr, followed by 1 μmol/L prednisone‐containing rats' serum for 48 hr (PDN + CTK). Each treatment was performed in triplicate.

### 2.5 Reverse transcription‐polymerase chain reaction (RT‐PCR)

Total RNA was extracted from cells using TRIzol reagent (Invitrogen, USA). cDNA was synthesized with PrimeScript RT reagent kit following the manufacturer's instructions. A standard protocol was used to perform RT‐PCR reactions on the Agilent StrataGene Mx3005 (Stratagene, USA). The primers sequences were as follows: GAPDH Forward: 5′‐ACTTGAAGGGTGGAGCCAAA‐3′ Reverse: 5′‐GCCCTTCCACAATGCCAAAG‐ 3′. Foxp3 Forward: 5′‐CTGGGGAAGCCATGGCAATA‐3′ Reverse: 5′‐TGAAGTAGGCGAACATGCGA‐3′. RORC Forward: 5′‐TGCGACTGGAGGACCTTCTA‐3′ Reverse: 5′‐AGACTGTGTGGTTGTTGGCA‐3′. IL17 Forward: 5′‐ACTACCTCAACCGTTCCACG‐3′ Reverse: 5′‐TTCCCTCCGCATTGACACAG‐3′. All experiments were run in duplicate with the same thermal‐cycling parameters.

### 2.6 Flow cytometry

After treatment for 72 hr, 2 × 10^6^ spleen lymphocytes were collected, washed with PBS and cultured in 1 ml of complete medium containing phorbol 12‐myristate 13‐acetate (PMA; 25 ng/ml), ionomycin (1 μg/ml) and Golgistop (2 μl, BD Pharmingen) for 5 hr. For labeling the Th17 cells, spleen lymphocytes were stained using PerCP/Cy5.5‐conjugated anti‐CD4 and PE‐conjugated anti‐human IL‐17. For Treg analysis, spleen lymphocytes were stained for PerCP‐Cy5.5‐conjugated anti‐CD4, APC‐conjugated anti‐CD25, and Alexa Fluor488‐conjugated anti‐CD127. All stained cells were counted via a BD flow cytometer with CellQuest software and analyzed using Flowjo software (Treestar, USA).

### 2.7 Enzyme‐linked immunosorbent assay (ELISA)

After treatment for 72 hr, the amount of culture medium was collected and centrifuged at 450 *g* for 5 min. The supernatant was collected to measure the concentrations of IL‐10, and IL‐17 by ELISA in accordance with the manufacturer's instructions. All samples were measured in triplicate. The detection ranges were as follows: 3.12 ~ 200 pg/ml (IL‐10), 93.75 ~ 6,000 pg/ml (IL‐17). The sensitivities of the ELISA kits were as follows: >0.78 pg/ml (IL‐10), >23.43 pg/ml (IL‐17).

### 2.8 Statistical analysis

Data are presented as *M* ± *SD*. Statistical analysis of differences between two groups was performed using the two‐sided Student's *t* test, while differences among more than two groups were analyzed using one‐way ANOVA followed. All statistical analyses were performed using SPSS 17.0 (IBM, Chicago, IL, USA). *p* < 0.05 was considered statistically significant.

## 3 RESULTS

### 3.1 Prednisone inhibited the mRNA expression of RORC and lymphocytes differentiation into Th17 cells

**Figure 1:**
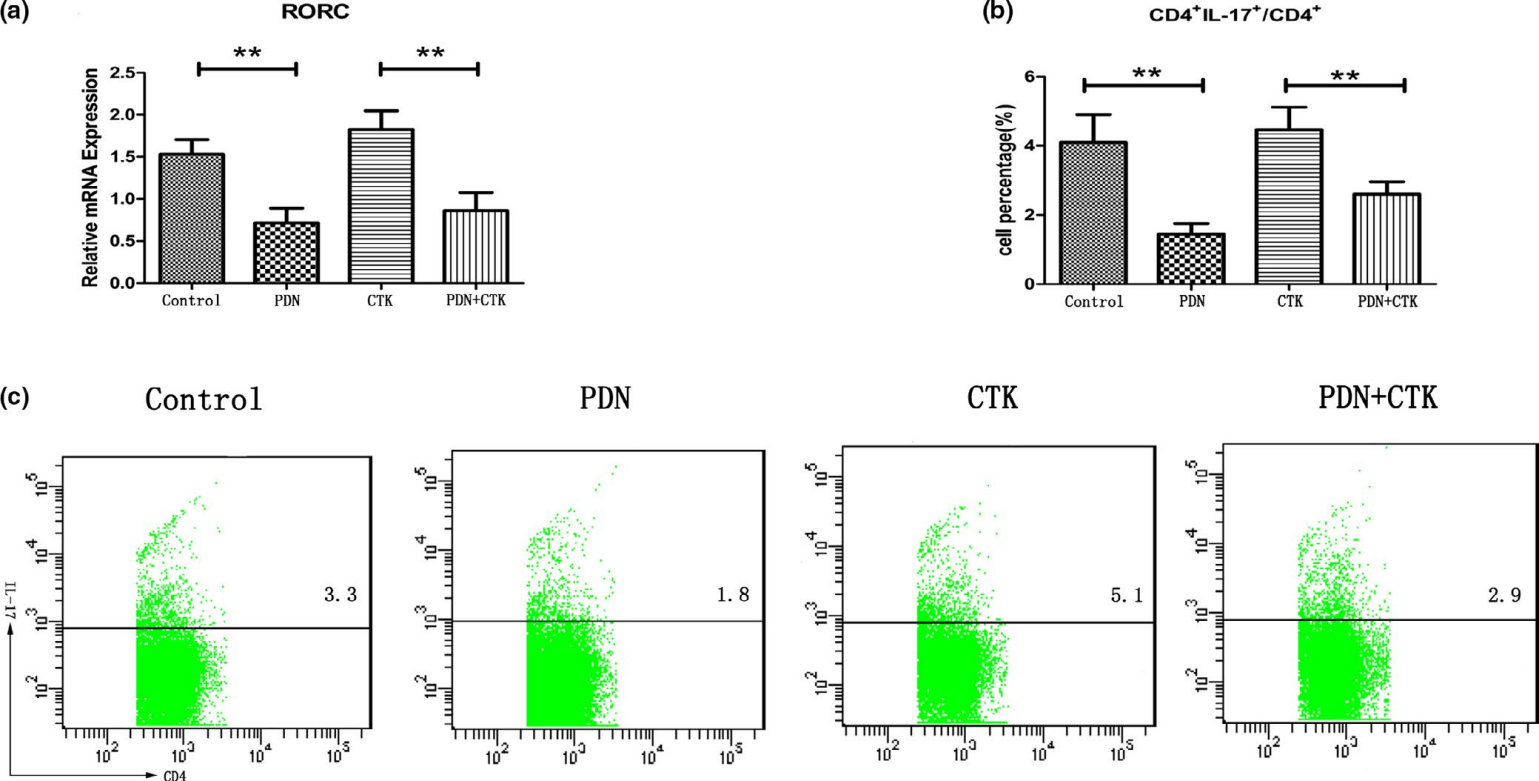
(a) The relative mRNA expression level of RORC in each group. (b) and (c) The percentages of Th17 (CD4^+ ^IL‐17^+^)/CD4^+^ in each group. **p < 0.01

Differentiation of CD4^+^ T cells into Th17 cells is controlled by a “master‐regulator” transcription factor, RORγt, which is a protein that in humans is encoded by the RORC (RAR‐related orphan receptor C) gene (Kimura & Kishimoto, 2011). Thus, in this part, we detected the relative amount of RORC mRNA expression in each group using RT‐PCR. It was observed that the mRNA expression of RORC in PDN group (0.72 ± 0.17) was significantly lower than that in control group (1.53 ± 0.17) (*p* < 0.01, Figure 1a) and the mRNA expression of RORC in PDN + CTK group (0.86 ± 0.21) was significantly lower than that in CTK group (1.83 ± 0.22) (*p* < 0.01, Figure 1a).

Flow cytometry was performed to test the quantity of the Th17 cells. Then the percentages of Th17 (CD4^+ ^IL‐17^+^)/CD4^+^ in different groups were compared. The percentage of Th17/CD4^+^ in PDN group (1.45 ± 0.30%) was significantly lower than that in control group (*p* < 0.01, Figure 1b,c). The percentage of Th17/CD4^+ ^cells in PDN + CTK group (2.60 ± 0.36%) was significantly lower than that in CTK group (4.47 ± 0.65%) (*p* < 0.01, Figure 1b,c).

This suggests that the methods to simulate the microenvironment of immunologic rejection by the effects of IL‐23, IL‐6, and TGF‐β on inducing the lymphocytes which were separated from the rats' spleens differentiation into Th17 cells are effective. Prednisone could obviously reduce the mRNA expression of RORC and inhibits the ability of proinflammatory cytokines to stimulate lymphocytes differentiation into Th17 cells.

### 3.2 Prednisone inhibited the mRNA expression of IL‐17 mRNA and the secretion of IL‐17

**Figure 2:**
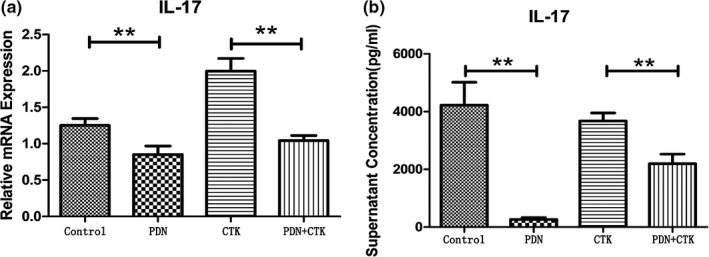
(a) The relative mRNA expression of IL‐17 in each group. (b) Concentrations of IL‐17 in each group. **p < 0.01

As the population of Th17 cells decreased, we wondered whether the secretion of these cells was also decreased. It is well known that IL‐17 is a key cytokine produced by Th17 cells (Dong, 2008). Thus RT‐PCR was performed to evaluate mRNA expression of IL‐17. The mRNA expression of IL‐17 in PDN group (0.85 ± 0.12) was significantly lower than that in control group (1.25 ± 0.10) (*p* < 0.01, Figure 2a) and the mRNA expression of IL‐17 in PDN + CTK group (1.04 ± 0.07) was significantly lower than that in CTK group (2.00 ± 0.17) (*p* < 0.01, Figure 2a). ELISA was performed to detect the concentrations of IL‐17 within the cell culture supernatant in the four groups. Concentration of IL‐17 in PDN group (269.52 ± 62.64 pg/ml) was significantly lower than that in control group (4,222.87 ± 795.18 pg/ml) (*p* < 0.01, Figure 2b) and the concentration of IL‐17 in PDN + CTK group (2,197.71 ± 332.20 pg/ml) was significantly lower than that in CTK group (3,677.29 ± 277.22 pg/ml) (*p* < 0.01, Figure 2b).

These results suggest prednisone may obviously reduce the mRNA expression of IL‐17. Prednisone can inhibit IL‐17A secretion, whereas IL‐23, IL‐6 and TGF‐β1 can stimulate IL‐17A secretion.

### 3.3 Prednisone enhanced the mRNA expression of Foxp3, induced differentiation of lymphocytes cells into Treg cells and enhanced the secretion of IL‐10

**Figure 3:**
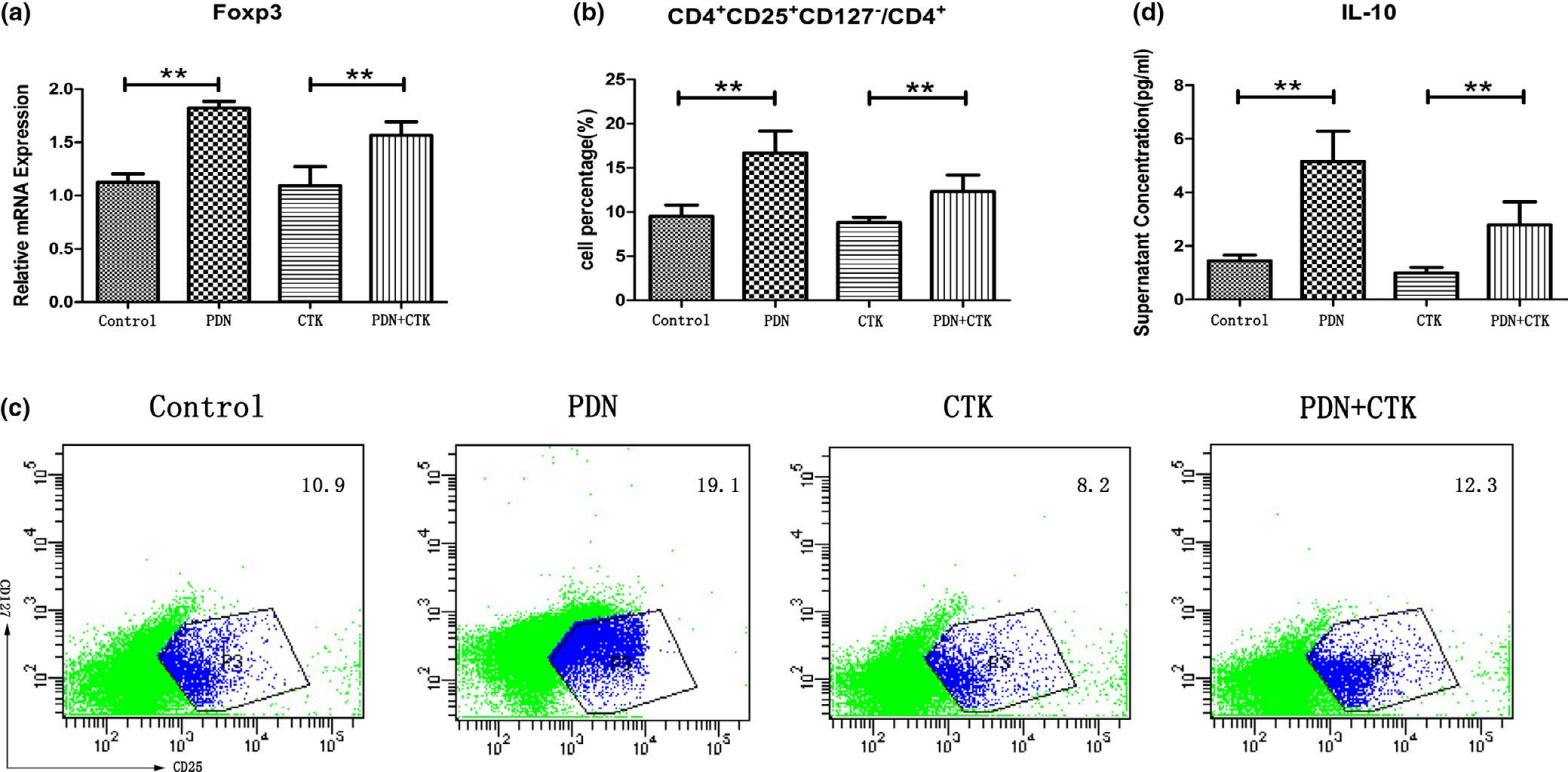
(a) The relative mRNA expression of Foxp3 in each group. (b) and (c) The percentages of Treg (CD4^+ ^CD25^+ ^CD127^−^)/CD4^+^ in each group. (d) Concentrations of IL‐10 in each group. **p < 0.01

The transcription factor Foxp3 is the most specific functional and phenotypic marker for Treg cells. Thus RT‐PCR was performed to detect the mRNA expression of Foxp3. The results showed that the mRNA expression of Foxp3 in PDN group (1.82 ± 0.06) was significantly higher than that in control group (1.12 ± 0.08) (*p* < 0.01, Figure 3a) and the mRNA expression of Foxp3 in PDN + CTK group (1.57 ± 0.13) was significantly higher than that in CTK group (1.09 ± 0.18) (*p* < 0.01, Figure 3a).

The percentages of Treg/CD4^+^ in the four groups were also tested by flow cytometry. Then the percentages of Treg (CD4^+ ^CD25^+ ^CD127^−^)/CD4^+^ in different groups were compared. The percentage of Treg/CD4^+^ in PDN group (16.70 ± 2.45%) was significantly higher than that in control group (9.53 ± 1.27%) (*p* < 0.01, Figure 3b,3). The percentage of Treg/CD4^+^ in PDN + CTK group (12.33 ± 1.85%) was significantly higher than that in CTK group (8.80 ± 0.60%) (*p* < 0.01, Figure 3b,c).

Treg cells at the maternal‐fetal interface are able to impede fetal rejection. This tolerant microenvironment is characterized by the expression of diverse immune‐modulating cytokines, such as IL‐10 and TGF‐β (Guerin, Prins, & Robertson, 2009). Thus the concentrations of IL‐10 of the culture supernatant in the four groups were detected to evaluate the functions of Treg cells (TGF‐β had been added to the culture media, thus we did not test it). Significant higher concentration of IL‐10 was detected in PDN group (5.16 ± 1.12 pg/ml), compared with control group (1.45 ± 0.21 pg/ml) (*p* < 0.01, Figure 3d) and higher concentration of IL‐10 was detected in PDN + CTK group (2.80 ± 0.86 pg/ml), compared with CTK group (1.00 ± 0.20 pg/ml) (*p* < 0.01, Figure 3d).

These results suggest that prednisone obviously enhanced the mRNA expression of Foxp3, induced the differentiation of lymphocytes into Treg cells and enhanced the secretion of IL‐10.

## 4 DISCUSSION

Most studies have suggested that prednisone modulates immune responses through different pathways. Low‐dose prednisone could inhibit the synthesis of those proinflammatory factors such as IL‐6, IL‐1, and TNF‐α (Cutolo, 2016). As the proliferation of immune cells decreases, the immune function is suppressed. Furthermore, prednisone prevents immunoglobulin binding with cell surface receptor; prevents the immune complexes passing through the basement membrane, and reduces the concentration of immunoglobulin and alexin. The traditional theory of cell model of Th1/Th2 which is insufficient to explain the mechanism of autoimmune diseases, maternal‐fetal immune tolerance and cancer immune reaction is gradually replaced by the Th17/Treg immune model. Therefore, we wondered if prednisone may have effect on Th17/Treg ratio to remodel the immune balance. The results of the current study suggested that the treatment with prednisone‐containing serum plays an important role in the modulation of immune responses in lymphocytes from rats' spleens.

The differentiation of lymphocytes into Th17 cells is initiated by signal transducer and activator of transcription 3 (STAT3), downstream of IL‐6‐ and IL‐21‐induced signaling. Activation of STAT3 induces the expression of RORα and RORγt. These two factors establish the Th17‐cell‐associated gene expression program, leading to the production of IL‐17A, IL‐17F and IL‐22. The key transcription factor, RORγt, is exclusively expressed in cells of the immune system. The expression of RORγt is induced by TGF‐β or IL‐6, and the overexpression of RORγt promoted lymphocytes differentiation into Th17‐cell. Previous studies have reported that patients with URSA appear to have a remarkably high amount of Th17 cells in the peripheral blood (Saifi et al., 2014) and decidua (Liu et al., 2011). After treated with methylprednisolone, patients with hepatitis B showed improvement with significant decreases in Th17 levels, and rebalanced Th17/Treg ratios (Lu et al., 2014). It was reported that the glucocorticoid receptor and STAT3 could interact with each other in a promoter‐dependent manner and the glucocorticoid receptor binding to DNA‐bound STAT3 inhibits STAT3 gene expression (Banuelos & Lu, 2016). As a result, the expression of RORγt was suppressed. In the current study, prednisone not only potentially inhibited RORC transcription, but also restrained generation of Th17 cells. IL‐17 may be reduced by suppression of Th17 cells or by glucocorticoids directly. It was observed in the current study that the mRNA expression level of IL‐17 was reduced after treated with prednisone, though IL‐6 which activated STAT3 was added in the culture media. These findings suggested that prednisone may resist the effect of proinflammatory factors.

Patterns of Foxp3^+^ Treg‐dependent immune suppression mediators include inhibition of proinflammatory cytokines such as interferon‐γ (IFN‐γ) and IL‐2 and suppression of T cells, dendritic cells, and macrophages through contact‐dependent and contact‐independent mechanisms. Sustained Foxp3 expression is critical for the capacity of Treg cells to negatively regulate the immune response. Besides, the expression of Foxp3 is tightly regulated by many transcription factors including STAT5 and all‐trans retinoic acid receptor (RAR). In the current study, we stained lymphocytes with CD4, CD25 and CD127 antibodies to identify the CD4^+^CD25^+^ Treg cells. It was demonstrated that CD127 expression, which inversely correlates with Foxp3, is an excellent biomarker of human Treg cells, especially when combined with CD25 (Liu, Putnam, Xu‐Yu, Szot, & Lee, 2006). A larger highly purified population of Treg cells could be obtained by a combination of CD4, CD25, and CD127, compared with the previous methods by other cell surface markers. In inflammatory bowel disease mice, there was a reduction of CD4^+^ T cells producing IFN‐γ, an increased frequency of the putative regulatory population of T cells producing IL‐10, and an increment in the frequency of the regulatory markers GITR, CTLA‐4, PD‐1, CD73, and FoxP3, after treating with glucocorticoid (Sales‐Campos et al., 2017). We have found that the serum concentration of IL‐10 in patients with URSA is much lower than that in healthy controls, whereas the expression of IL‐17 is at a higher level (Cai, Li, Huang, Fu, & Wu, 2016). It was reported by a recent study that the patients with URSA who received immunotherapy got an increment of Treg cells and an reduction of IL‐2 level. As a result of this, pregnancy success rates of those patients were significantly higher (Chen, Yang, Huang, & Li, 2016). Similarly, higher proportion of Treg cells and a higher level of IL‐10 were also observed after treated with prednisone in this research. It prompted that prednisone which contributes to the restoration of immune balance could be used in some patients with URSA which was caused by the abnormal immune system.

In conclusion, our results suggest that prednisone‐contained serum may induce the lymphocytes from female rats' spleens differentiation into Treg cells as well as inhibit them differentiation into Th17 cells, accompanied by a reduction of IL‐17 secretion and an increment of IL‐10 secretion. Thus we assume that prednisone may rebuild the immunologic homeostasis and may be to use in human diseases with changes in the imbalance immune system, such as URSA, hepatitis B infection or other autoimmune diseases.

## CONFLICTS OF INTEREST

The authors declare no conflicts of interest.

## AUTHOR CONTRIBUTIONS

Mu‐Jun Li designed the study. Xiao‐Qian Fu and Jun‐Ying Cai performed the research. Xiao‐Qian Fu performed the statistical analyses. Xiao‐Qian Fu and Jun‐Ying Cai wrote the manuscript. All authors reviewed the manuscript.
